# Quality of testicular spermatozoa improves with changes in composition of culture medium

**DOI:** 10.1186/s12610-023-00198-8

**Published:** 2023-09-07

**Authors:** Lida Gholizadeh, Mohammad Ali Khalili, Behnam Maleki, Serajoddin Vahidi, Azam Agha-Rahimi

**Affiliations:** 1 Research and Clinical Center for Infertility, Yazd Reproductive Sciences Institute, Shahid Sadoughi University of Medical Sciences, Yazd, Iran; 2 Infertility Center, Mazandaran University of Medical Sciences, Sari, Iran; 3 Andrology Research Center, Yazd Reproductive Sciences Institute, Shahid Sadoughi University of Medical Sciences, Yazd, Iran

**Keywords:** Azoospermia, Artificial seminal fluid, Mitochondrial membrane potential, DNA fragmentation index, Azoospermie, Liquide séminal artificiel, Potentiel de Membrane mitochondriale, Indice de Fragmentation de l'ADN

## Abstract

**Background:**

Spermatozoa retrieved from the testis and epididymis are deprived of the beneficial effects of seminal fluid. Thus applying an artificial medium with normal seminal fluid characteristics, known as artificial seminal fluid (ASF), may provide an appropriate condition for improving some sperm parameters in azoospermia. The objective was to investigate the impact of in vitro exposure of testicular and epididymal spermatozoa to ASF on sperm quality. The study was conducted on testicular (*n* = 20) and epididymal (*n* = 20) sperm specimens obtained from azoospermic men. Each sample was divided into two equal parts: Part I) for processing and incubation with Ham’s F10 medium; Part II) for processing and incubation with ASF.

**Results:**

After 2 h incubation, testicular sperm motility was significantly higher in ASF than in Ham’s F10 medium. In comparison to 0 h, mitochondrial membrane potential levels of testicular spermatozoa were significantly higher after 2 h and 24 h in ASF and after 24 h in Ham’s F10 medium. Furthermore, the data indicated significantly lower rates of epididymal spermatozoa with high MMP in both media after 24 h. There were no significant differences in the DNA fragmentation index of testicular and epididymal spermatozoa between ASF and Ham’s F10 medium at different time points.

**Conclusion:**

The results demonstrated that in vitro incubation of testicular spermatozoa improved their motility more effectively than Ham’s F10 medium in the short term (2 h), but had no effect on epididymal spermatozoa. Since the physiology of testicular spermatozoa is different from that of ejaculated spermatozoa, it seems that a special environment should be designed and used for each of them.

## Résumé

**Contexte:**

Les spermatozoïdes prélevés dans les testicules et les épididymes sont privés des effets bénéfiques du liquide séminal. Ainsi, l'utilisation d'un milieu artificiel avec des caractéristiques normales du liquide séminal, connu sous le nom de liquide séminal artificiel (ASF), peut constituer une condition appropriée pour améliorer certains paramètres des spermatozoïdes obtenus dans l'azoospermie. L'objectif était d'étudier l'impact de l'exposition in vitro de spermatozoïdes testiculaires et épididymaires à l’ASF sur la qualité du sperme. L'étude a été menée sur des échantillons de spermatozoïdes testiculaires (*n* = 20) et épididymaires (*n* = 20) obtenus chez des hommes azoospermiques. Chaque échantillon a été divisé en deux parties égales: Partie I) pour le traitement et l'incubation avec le milieu F10 de Ham; Partie II) pour la transformation et l'incubation avec l’ASF.

**Résultats:**

Après 2 h d'incubation, la mobilité des spermatozoïdes testiculaires était significativement plus élevée dans l’ASF que dans le milieu F10 de Ham. Par rapport à 0 h, les niveaux du potentiel de membrane mitochondriale (PMM) des spermatozoïdes testiculaires étaient significativement plus élevés après 2 h et 24 h dans l’ASF, et après 24 h dans le milieu F10 de Ham. En outre, les données ont indiqué des taux significativement plus faibles de spermatozoïdes épididymaires avec un PMM élevé dans les deux milieux après 24 heures. Il n'y avait pas de différences significatives dans l'indice de fragmentation de l'ADN des spermatozoïdes testiculaires et épididymaires entre l’ASF et le milieu F10 de Ham aux différents temps.

**Conclusion:**

Les résultats ont montré que l'incubation in vitro de spermatozoïdes testiculaires dans l’ASF améliorait leur mobilité plus efficacement que le milieu F10 de Ham à court terme (2 h), mais n'avait aucun effet sur les spermatozoïdes épididymaires. Étant donné que la physiologie des spermatozoïdes testiculaires est différente de celle des spermatozoïdes éjaculés, il semble qu'un environnement spécial devrait être conçu et utilisé pour chacun d'eux.

## Background

Selection of viable, high-quality spermatozoa is critical for improving the assisted reproductive technology (ART) outcome in azoospermic men [1]. Owing to the poor motility of testicular and occasionally epididymal spermatozoa, identification of viable spermatozoa for intracytoplasmic sperm injection (ICSI) is difficult, resulting in low fertilization rates [2]. In general, testicular spermatozoa are physiologically more immature than epididymal or ejaculated spermatozoa, and are often immotile or show only twitching motility immediately after biopsy [3]. In vitro incubation of spermatozoa in various media could be considered as an approach for the stimulation of motility of hypokinetic cells, although no consensus has yet been reached on the optimal conditions for this procedure [4]. It has been demonstrated that some characteristics of seminal fluid (SF) have a direct influence on sperm quality [5, 6].

SF is an important constituent of semen that has a crucial role in sperm metabolism, function, survival, motility and maturation [7]. SF is composed of a variety of macro and trace elements, such as Ca^2+^, Mg^2+^, Zn^2+^, K^+^, Na^+^, and Cl^−^, which play significant roles in sperm quality and function. For example, Ca^2+^ is related to sperm motility, metabolism, the acrosome reaction and fertilizing capacity [8]. Zn^2+^ is involved in antioxidant reactions influencing sperm motility [9]; Mg^2+^ is an essential cofactor in enzymatic reactions involving energy metabolism and nucleic acid synthesis [10].

Since semen forms during the process of ejaculation, the testicular and epididymal spermatozoa collected in situ are deprived of the beneficial effects of SF. As a result, the use of these spermatozoa in assisted reproduction may generate lower fertilization rates than ejaculated spermatozoa. Given the importance of SF in the final maturation of human spermatozoa, we hypothesize that SF has certain positive effects on surgically-retrieved spermatozoa. However, the exchange of biological SF between human specimens is not a safe strategy in ART, because of the high risk of contaminating the semen specimen with donor’s spermatozoa; thus, it appears preferable to utilize an artificial medium with normal SF characteristics.

We previously designed a culture medium with biochemical characteristics of semen, named artificial seminal fluid (ASF), and reported its preservative effects as a cryoprotectant in the process of human sperm vitrification [11]. Furthermore, we recently demonstrated the beneficial effects of ASF on sperm motility of asthenozoospermic ejaculates during the incubation period [12]; however, there are no reports evaluating the effect of ASF on testicular and epididymal spermatozoa. The current study aimed to determine the impact of in vitro exposure of testicular and epididymal spermatozoa to ASF on sperm quality in azoospermic men.

## Materials and methods

### Participants and sample collection

The study included testicular (*n* = 20) and epididymal (*n* = 20) sperm specimens from men diagnosed with azoospermia who were referred to our Andrology Laboratory. The methods used for diagnosis the type of azoospermia were endocrine profile, clinical examination, semen analysis and genetic tests. Sperm specimens were obtained from the patients via percutaneous epididymal aspiration (PESA) or testicular sperm extraction (TESE) methods. No epididymal and testicular samples originated from the same person. The presence of at least 50 spermatozoa in each specimen, and men aged up to 40 years, were our inclusion criteria. Men with a history of varicocele, drug abuse, or heavy smoking were excluded from the study. The samples were obtained from men with epididymitis (*n* = 6), spermatic cord torsion (*n* = 3), failed vasectomy reversal (*n* = 8), spinal cord injury (*n* = 3), Bilateral herniorrhaphy (*n* = 5), zinner syndrome (*n* = 1), and idiopathic azoospermia (*n* = 14). Clinical characteristics of the participants are demonstrated in Table 1.

**Table 1:** Clinical characteristics of the study participants

Clinical characteristics	Testicular spermatozoa	Epididymal spermatozoa
Type of infertility		
Primary	*n* = 7	*n* = 5
Secondary	*n* = 13	*n* = 15
Type of azoospermia		
Obstructive	*n* = 17	*n* = 20
Non-obstructive	*n* = 3	*n* = 0

### Experimental design

**Fig. 1:**
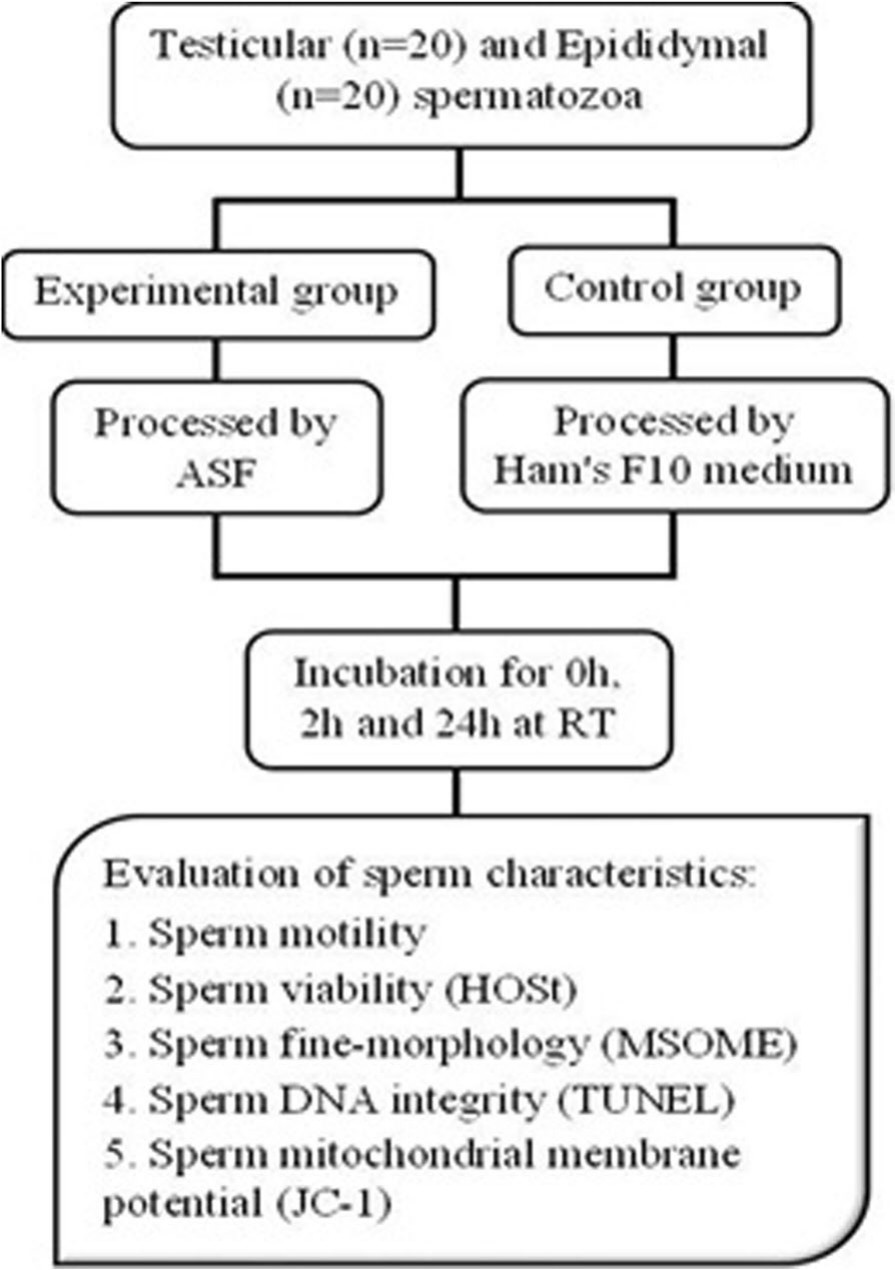
Schematic diagram for sample collection and study design. Abbreviations: ASF: artificial seminal fluid; RT: room temperature; HOSt: hypo-osmotic swelling test; MSOME: motile sperm organelle morphology examination; TUNEL: terminal deoxynucleotidyl transferase dUTP nick end labeling

The sperm samples were collected and transferred to the laboratory for processing and microscopic examination. Each specimen was divided into two equal parts: Part I) for processing with Ham’s F10 medium without HEPES + 5 mg/mL human serum albumin (HSA). The harvested pellet was diluted with Ham’s F10 medium (Control); Part II) for processing with ASF + 5 mg/mL HSA. The pellet was diluted with ASF (Experimental group). Each combination of samples was kept in a test tube and incubated for 0 h, 2 h, and 24 h at room temperature (RT). The sperm motility, viability, fine morphology, DNA and mitochondrial integrity were assessed immediately after processing (0 h), as well as 2 h and 24 h after incubation to investigate the effects of in vitro incubation with ASF and Ham’s F10 medium on sperm quality. All analyses were performed in a blinded way. The schematic diagram for the study design is illustrated in Fig. 1.

### Artificial seminal fluid (ASF)

The ASF components were as follows: NaCl: 2.69 g/L; sodium citrate 2H_2_O: 8.13 g/L; KCl: 0.432 g/L; K_2_HPO_4_: 1.91 g/L; Na pyruvate: 0.374 g/L; Na lactate: 0.779 g/L; glucose.1H_2_O: 1.12 g/L; fructose: 2.72 g/L; NaHCO_3_: 2.1 g/L; sodium urate: 0.07 g/L; urea: 0.72 g/L; MgSO_4_: 0.54 g/L; ZnSO_4_.7H_2_O: 0.5 g/L; CaCl_2_.2H_2_O: 0.73 g/L; gentamycin: 40 mg/L; and distilled water. The ASF osmolality and pH were adjusted to 325 ± 10 mosmol/L and 7.4 respectively [11]. All materials were purchased from Sigma-Aldrich (St. Louis, MO, USA).

### Assessment of sperm motility and viability

Motility of testicular and epididymal spermatozoa was evaluated by using an inverted microscope at × 400 magnification. The presence of any sign of movement or twitching indicated motility. The assessment of sperm vitality was performed by a modified HOS (hyp-osmotic solution) test. For this, the spermatozoa were transferred from their original separate micro-droplets containing ASF + 5 mg/mL HSA and Ham’s F10 medium + 5 mg/mL HSA into micro-droplets of hypo-osmotic medium prepared by mixing ASF and Ham’s F10 medium with an equal amount of deionized-grade water [13]. The spermatozoa were exposed to HOS solution for 5 to 10 s. After a maximum of 10 s in HOS solution, a viable spermatozoon was recognized by its curved or swollen tail.

### Assessment of fine sperm morphology

The Motile Sperm Organelle Morphology Examination (MSOME) technique was applied for evaluation of fine sperm morphology. Spermatozoa were transferred to micro-droplets of ASF + 5 mg/mL HSA or Ham’s F10 medium + 5 mg/mL HSA placed in a sterile glass-bottom dish under paraffin oil. The spermatozoa were then examined at high magnification (× 6600) using an inverted microscope outfitted with high-power differential interference contrast optics. The spermatozoa were classified into three groups of high, medium and low-quality based on the head shape, presence of vacuoles, and the shape of the head base according to Cassuto and Barak’*s* classification [14].

### Assessment of sperm DNA integrity

The sperm DNA fragmentation was assessed by TUNEL assay using In Situ Cell Death Detection kit (Roche Diagnostics GmbH, Mannheim, Germany) [15]. Briefly, the cells were transferred to 5 µL phosphate-buffered saline droplets on a glass slide and air-dried. The slides were then fixed and incubated with blocking solution (3% H_2_O_2_ in 99.8% methanol) in a humid, dark chamber for 20 min. After cell permeabilization with 0.1% Triton X-100, the slides were incubated with the TUNEL reaction mixture (50 μL) in a humid, dark chamber at 37ºC for 1 h. The slides were then stained with 50 μL converter-POD at 37ºC for 1 h, followed by exposure to the 3,3’-diaminobenzidine tetrahydrochloride (DAB) substrate solution in a dark chamber at 37ºC for 20 min. After dehydration in serial ethanols (70%, 90% and 100%), the slides were evaluated by light microscopy at × 1000 magnification. The spermatozoa with dark brown heads were considered to have fragmented DNA (TUNEL^+^), and those with either pale or light brown heads were counted as the cells without DNA fragmentation (TUNEL^−^). Finally, the DNA fragmentation index (DFI), as one of the indicators of DNA damage, was calculated.

### Assessment of sperm mitochondrial membrane potential

The level of sperm mitochondrial membrane potential (MMP) was determined by using a JC-1 mitochondrial membrane potential assay kit (Cayman Chemical Company, Ann Arbor, MI, USA). In brief, JC-1 staining solution was prepared by diluting the stock solution (1:10 v/v) in the sperm washing medium according to the manufacturer’s instructions. JC-1 working solution was prepared by mixing 5 μL of staining solution with sperm washing medium (final volume: 150 μL). For each analysis, the spermatozoa were transferred to 5 μL of JC-1 working solution using the ICSI needle equipped with a micromanipulator. After 30 min of incubation at 37 °C in the dark, cells were evaluated by a fluorescence microscope at × 1000 magnification [16]. Spermatozoa with high MMP fluoresce red and the sperm midpiece appears yellow or orange. In contrast, sperm with low MMP fluoresce green. The percentage of spermatozoa with a yellow or orange stained mid-piece was considered to have a high mitochondrial membrane potential.

### Statistical analysis

The Kolmogorov–Smirnov test was used to determine the distribution of values for each parameter. Statistical significance was assessed using One-Way Repeated-Measures ANOVA test. The significance level was set at *P* < 0.05. All analyses and plotted graphs were performed with GraphPad Prism 8.4.2 (GraphPad Software, Inc., San Diego, CA, USA). Data were represented as box and whisker plots, whereby boxes depict the 25th and 75th percentiles with a horizontal line inside the box depicting the median value, and whiskers depict the 10th and 90th percentiles.

## Results

### Testicular spermatozoa after exposure to the tested media

#### Sperm parameters

A total of 8107 spermatozoa was assessed for testicular sperm motility. Our result showed that after 2 h of incubation, the motility of testicular spermatozoa was significantly higher in ASF than in Ham’s F10 medium (*P* = 0.008). We also found that over time, the motility of testicular spermatozoa was significantly higher in ASF after both 2 h and 24 h of incubation than at 0 h (*P* = 0.009 and *P* < 0.001, respectively), whereas in Ham’s F10 medium, a significant increase in the motility was observed only after 24 h of incubation (*P* < 0.001).

To determine the testicular sperm viability and fine-morphology, 3780 spermatozoa were examined. We detected no significant changes in the rates of viable spermatozoa in ASF after 24 h, whereas the incubation in Ham’s F10 medium for 24 h was accompanied by a significant decline in sperm viability compared to that immediately after processing (0 h) (*P* = 0.03).

**Fig. 2:**
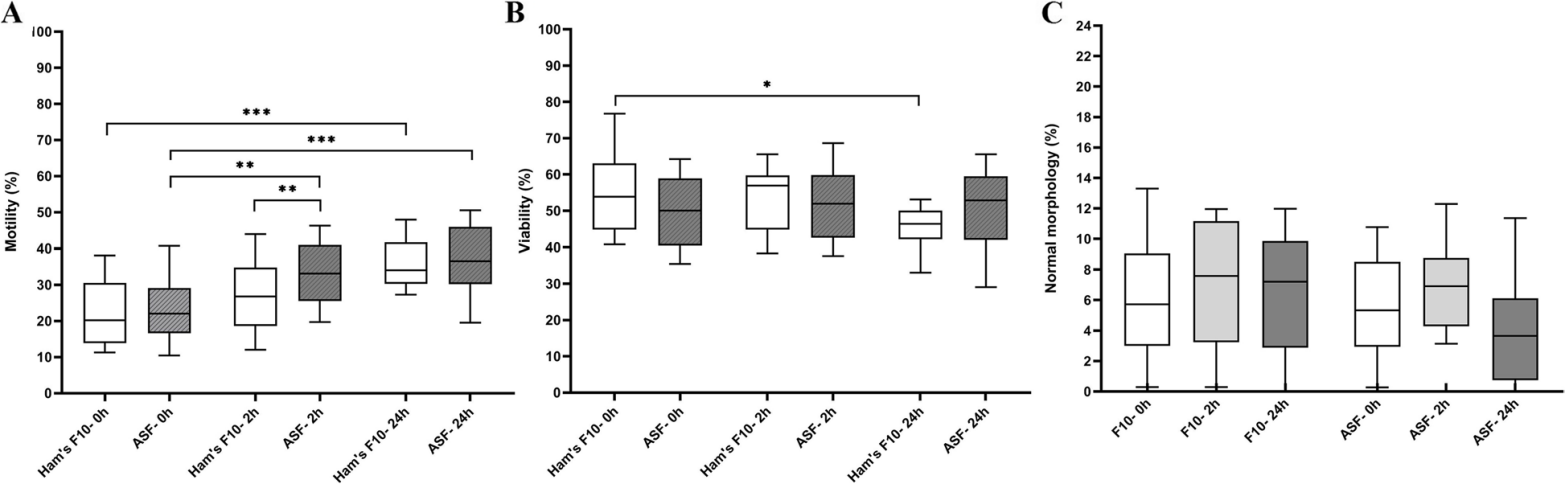
**A** Testicular sperm motility (%) in tested media at different time points. **B** Testicular sperm viability (%) in tested media at different time points. **C** Testicular sperm fine morphology (%) in tested media at different time points. The boxes depict the 25th and 75th percentiles with a horizontal line inside the box depicting the median value, and whiskers depict the 10th and 90th percentiles. One-Way Repeated-Measures ANOVA test. *P < 0.05, **P < 0.01, ***P < 0.001. Abbreviations: ASF: artificial seminal fluid

Moreover, our data demonstrated the similar proportions of high-quality spermatozoa between ASF and Ham’s F10 medium at all-time points. The details are shown in Fig. 2.

#### Sperm mitochondrial integrity

**Fig. 3:**
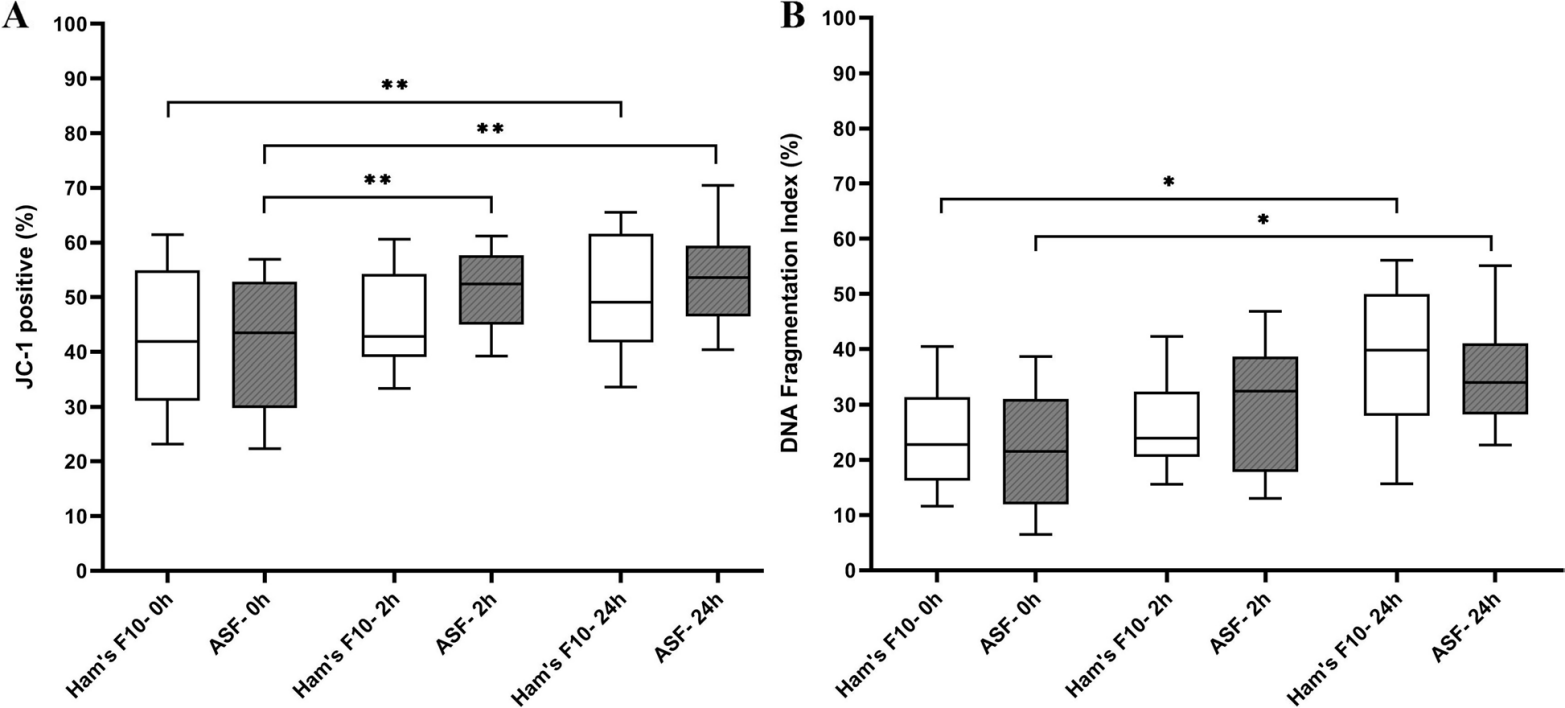
**A** Testicular sperm mitochondrial membrane potential (%) in tested media at different time points. **B** Testicular sperm DNA fragmentation index (%) in tested media at different time points. The boxes depict the 25th and 75th percentiles with a horizontal line inside the box depicting the median value, and whiskers depict the 10th and 90th percentiles. One-Way Repeated-Measures ANOVA test. *P < 0.05, **P < 0.01. Abbreviations: ASF: artificial seminal fluid

For assessment of MMP, 3093 spermatozoa were analyzed. The proportion of spermatozoa with high MMP (JC-1^+^) was not significantly different in ASF from that in Ham’s F10 medium after 2 h and 24 h. The MMP of testicular spermatozoa was significantly increased in ASF after 2 h and 24 h compared with that immediately after sperm processing (*P* = 0.008 and *P* = 0.009, respectively); while a significant increase in MMP was observed in Ham’s F10 medium only after 24 h (*P* = 0.005). The details are shown in Fig. 3.

#### Sperm DNA integrity

To determine the testicular sperm DFI, 2741 spermatozoa were evaluated. As it is shown in Fig. 3, there were no significant differences in the percentages of testicular sperm DFI between ASF and Ham’s F10 medium after the 2 h and 24 h time points. However, the percentage of testicular spermatozoa with fragmented DNA was significantly increased in both ASF and Ham’s F10 medium after 24 h, when compared with that immediately after processing (0 h) (*P* = 0.01 and *P* = 0.02, respectively).

### Epididymal spermatozoa after exposure to the tested media

#### Sperm parameters

A total of 9645 spermatozoa were assessed for epididymal sperm motility. The percentages of viable spermatozoa were similar between ASF and Ham’s F10 medium in epididymal specimens at all-time points. After 24 h incubation, the motility of epididymal spermatozoa was reduced in both ASF and Ham’s F10 medium (*P* = 0.01), whereas no significant differences were noticed in sperm motility after 2 h.

**Fig. 4:**
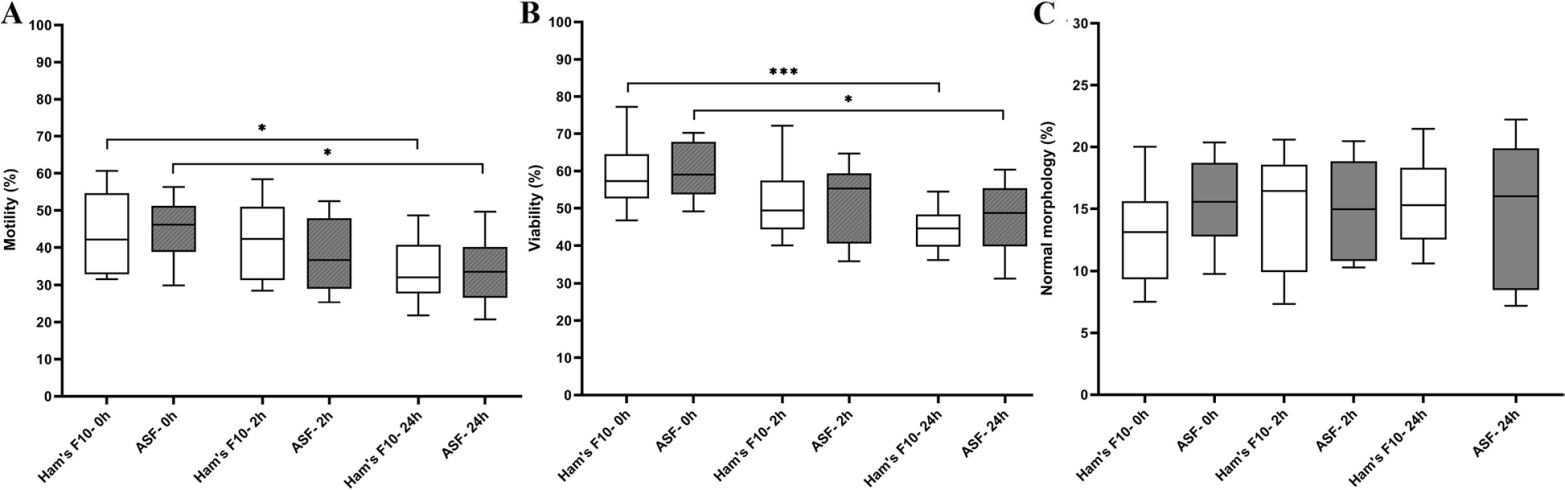
**A** Epididymal sperm motility (%) in tested media at different time points. **B** Epididymal sperm viability (%) in tested media at different time points. **C** Epididymal sperm fine morphology (%) in tested media at different time points. The boxes depict the 25th and 75th percentiles with a horizontal line inside the box depicting the median value, and whiskers depict the 10th and 90th percentiles. One-Way Repeated-Measures ANOVA test. *P < 0.05, ***P < 0.001. Abbreviations: ASF: artificial seminal fluid

To determine the epididymal sperm viability and fine-morphology, 4788 spermatozoa were examined. The data demonstrated that sperm viability was significantly decreased in both ASF and Ham’s F10 medium after 24 h of incubation (*P* = 0.01 and *P* = 006, respectively). Similar proportions of high-quality spermatozoa were detected in ASF and Ham’s F10 medium at all-time points. The details are shown in Fig. 4.

#### Sperm mitochondrial integrity

**Fig. 5:**
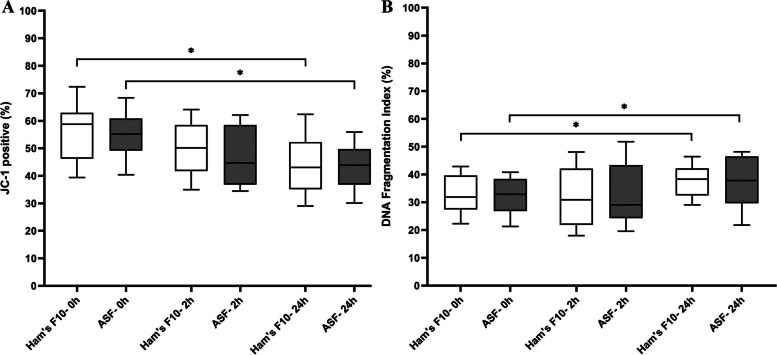
**A** Epididymal sperm mitochondrial membrane potential (%) in tested media at different time points. **B** Epididymal sperm DNA fragmentation index (%) in tested media at different time points. The boxes depict the 25th and 75th percentiles with a horizontal line inside the box depicting the median value, and whiskers depict the 10th and 90th percentiles. One-Way Repeated-Measures ANOVA test. *P < 0.05. Abbreviations: ASF: artificial seminal fluid

For assessment of MMP, 3687 spermatozoa were analyzed. The proportion of spermatozoa with high MMP (JC-1^+^) was not significantly different between ASF and Ham’s F10 medium at different time points. The data showed significantly lower percentages of epididymal spermatozoa with high MMP in both ASF and Ham’s F10 medium after 24 h than immediately after processing (0 h) (*P* = 0.01). The details are shown in Fig. 5.

#### Sperm DNA integrity

To determine the epididymal sperm DFI, 3805 spermatozoa were evaluated. There were no significant differences in the proportion of epididymal sperm DFI between ASF and Ham’s F10 medium at different time points. The percentage of epididymal spermatozoa with fragmented DNA was significantly increased in both ASF and Ham’s F10 medium after 24 h, compared with immediately after processing (0 h) (*P* = 0.01 and *P* = 0.03, respectively).

## Discussion

Although a small improvement in sperm motility may not be clinically significant in a normal patient group, a small increase in motility would facilitate the selection of viable spermatozoa with normal morphology in the severe male factor subgroup, including azoospermia. This would undoubtedly enhance the number of oocytes inseminated and improve the chances of fertilization.

In the current study, a remarkable increase in the motility of testicular spermatozoa was observed after in vitro incubation for short (2 h) and long (24 h) periods, although the impact of Ham’s F10 medium on motility was revealed only after long-term incubation. The present findings are consistent with other studies that confirmed improvement in testicular sperm motility after in vitro incubation under various conditions [17–20].

Co-culturing with all testicular cells, including Sertoli, Leydig and germ cells [21] and removing inhibitory factors with a simple wash before incubation [22] have been proposed as factors for sperm motility enhancement. We also assumed that high MMP of spermatozoa may be considered a key factor for motility improvement. The previous studies displayed that MMP, which is a direct reflection of the amount of ATP produced, has a strong relationship with functional sperm parameters such as motility [23–25]. In our study, a remarkable increase in the percentage of testicular spermatozoa with high MMP was detected in both media over time.

According to our findings, ASF improved testicular sperm motility more effectively than Ham’s F10 medium in short term (2 h). One explanation for this could be the presence of many energy substrates in ASF. Our artificial medium contains the substrates such as glucose, fructose, pyruvate and lactate as energy sources, on which sperm motility is mainly dependent, whereas Ham’s F10 medium only has glucose and pyruvate [26]. In addition, a negative correlation between semen levels of fructose, as the main source of energy for the sperm motility has been confirmed, especially in in vitro conditions [27].

Moreover, it is well established that spermatozoa contain several ATPases, each of which is dependent on the different cations such as Na^+^, K^+^, and Mg^2+^, that are responsible for breaking down ATP to release energy for flagellar contractile processes [28]. Therefore, higher values of these ions in ASF than in Ham’s F10 could explain the higher sperm motility in this medium. Considering that ASF is relatively rich in hyperactivation prerequisites, including ions and some biochemical compounds, this medium appears to be more effective than Ham’s F10 in stimulating or inducing activation in spermatozoa.

In contrast to testicular spermatozoa, our data showed a significant drop in the epididymal sperm motility across tested media, especially after long-term (24 h) incubation. Accordingly, Edirisinghe and colleagues reported that during in vitro culture of epididymal spermatozoa, the progressive motility declined gradually during the incubation period [20]. Since epididymal spermatozoa are deprived of the possible benefits of the co-culturing system suggested for testicular specimens, this may be considered a reason for the decrease of motility in these samples after in vitro incubation. This decline could be also related to the changes in MMP of epididymal spermatozoa during incubation, as a decrease in the percentage of epididymal spermatozoa with high MMP was observed over time in this study. On the basis of previous evidence and our findings, in vitro incubation does not appear to be very useful for epididymal spermatozoa. It is known that epididymal spermatozoa are more mature than testicular ones. The benefits of in vitro incubation seem to be greater in the case of spermatozoa which are inherently very immature.

Successful fertilization, normal embryo development, and pregnancy, as well as accurate transmission of paternal genetic material to offspring, all require sperm chromatin/DNA integrity [29]. In this study, ASF and Ham’s F10 medium functioned similarly in terms of preserving sperm DNA integrity during the incubation period in both the testicular and epididymal specimens. We also recorded a significant increase in the percentage of spermatozoa with fragmented DNA in both the media after 24 h. There is increasing evidence that long-term incubation of spermatozoa causes spontaneous DNA damage and alters chromatin status. Our results are in agreement with other studies that found a time-dependent increase in sperm DNA fragmentation [30–32]. In addition, it has been reported that DNA fragmentation in testicular spermatozoa measured with the TUNEL assay increases after 4 h and 24 h of incubation, with the rate of fragmentation being higher after long incubation than short [33]. It is believed that long-term sperm in vitro incubation is associated with sperm ageing. Because of the high presence of unsaturated fatty acids in their membrane and a lack of antioxidants in their cytoplasm, spermatozoa are vulnerable to harmful effects of reactive oxygen species (ROS), which may result in sperm DNA damage. Several reports suggest that spermatozoa produce ROS, which is responsible for causing negative effects in spermatozoa during in vitro incubation, including DNA damage [34, 35].

The evaluation of fine sperm morphology at high magnification using the MSOME procedure may be a useful tool for sperm selection in ART [36]. It is still unclear whether specific in vitro conditions during sperm preparation and manipulation result in changes in the fine morphology of spermatozoa. With respect to our finding on MSOME procedure, it is assumed that both ASF and Ham’s F10 medium had similar effects in maintaining sperm morphology. In addition, it could be inferred that both media could be good options for the short and long-term in vitro incubation of both the testicular and epididymal spermatozoa because the rate of high-quality spermatozoa which is the preferred quality of sperm for ICSI was not affected.

By considering the significant effects of biological SF on sperm metabolism, nutrition, function as well as final maturation of human spermatozoa, applying an artificial medium with normal SF characteristics may provide a suitable condition for improvement of some sperm parameters in ART laboratories. We designed a medium with the chemical properties of human SF, with a formulation containing all the major ions of biological SF with the same osmolality. The only protein present in our SF formulation was serum albumin. Adding some amino acids and protein compounds to ASF may help to adjust its osmolality as much as possible. Our ASF formulation focused largely on ion compositions of biological SF. Although ASF has some components with antioxidant capacities, such as urea, albumin, zinc, and pyruvate, it lacks other biological SF antioxidants. Thus, the enrichment of ASF with these components may help to generate an ideal culture medium for the preservation of sperm quality during the incubation period, especially in long term. This study demonstrates that the composition of the culture medium can affect testicular sperm parameters. Since the physiology of testicular spermatozoa is different from that of ejaculated spermatozoa, it seems that a special environment should be designed and used for each of them. We acknowledge some limitations of the present study. A major limitation is that, owing to ethical considerations, it was not possible to utilize spermatozoa incubated in ASF for clinical purposes to investigate the outcomes, such as the rate of fertilization, embryo development, pregnancy, and live birth. Moreover, the majority of testicular and epidydimal samples (65% and 75%, respectively) were obtained from azoospermic men with secondary infertility, which may impress the generalization of the finding to all azoospermic cases.

## Conclusion

The findings confirmed that the in vitro incubation of testicular spermatozoa for up to 24 h significantly improved their motility. In the short term (2 h), ASF enhanced testicular sperm motility more effectively than Ham’s F10 medium, despite not improving epididymal spermatozoa.

## Data Availability

The datasets used and analysed during the current study are available from the corresponding author on reasonable request.

## Abbreviations

ART: Assisted reproductive technology
ICSI: Intracytoplasmic sperm injection
SF: Seminal fluid
ASF: Artificial seminal fluid
PESA: Percutaneous epididymal aspiration
TESE: Testicular sperm extraction
HSA: Human serum albumin
HOS: Hypo-osmotic swelling
MSOME: Motile sperm organelle morphology examination
DFI: DNA fragmentation index
MMP: Mitochondrial membrane potential
ROS: Reactive oxygen species
